# Gitelman Syndrome in a School Boy Who Presented with Generalized Convulsion and Had a R642H/R642W Mutation in the *SLC12A3* Gene

**DOI:** 10.1155/2014/279389

**Published:** 2014-07-16

**Authors:** Shigeru Makino, Toshihiro Tajima, Jun Shinozuka, Aki Ikumi, Hitoshi Awaguni, Shin-ichiro Tanaka, Rikken Maruyama, Shinsaku Imashuku

**Affiliations:** ^1^Division of Pediatrics, Uji-Tokushukai Medical Center, Uji, Kyoto, Japan; ^2^Department of Pediatrics, Hokkaido Graduate School of Medicine, Sapporo, Japan; ^3^Division of Laboratory Medicine, Uji-Tokushukai Medical Center, Uji, Kyoto 611-0042, Japan

## Abstract

An 8-year-old Japanese boy presented with a generalized convulsion. He had hypokalemia (serum K 2.4 mEq/L), hypomagnesemia, and metabolic alkalosis (BE 5.7 mmol/L). In addition, his plasma renin activity was elevated. He was tentatively diagnosed with epilepsy on the basis of the electroencephalogram findings and was treated by potassium L-aspartate and carbamazepine to control the hypokalemia and seizure, respectively. However, a year later, the patient continued to have similar abnormal laboratory data. A presumptive diagnosis of Gitelman syndrome (GS) was then made and the patient's peripheral blood mononuclear cells were subjected to sequence analysis of the *SLC12A3* gene, which encodes a thiazide-sensitive sodium-chloride cotransporter. The patient was found to have compound heterozygous mutations, namely, R642H inherited from his father and R642W inherited from his mother. Thus, if a patient shows persistent hypokalemia and metabolic alkalosis, GS must be considered, even if the patient exhibits atypical clinical symptoms.

## 1. Introduction

Gitelman syndrome (GS) is characterized with hypokalemia, metabolic alkalosis, hypocalciuria, and hypomagnesemia [1]. It occurs in older children or adults and is often confused with surreptitious diuretic abuse, laxative abuse, or Bartter's syndrome [2]. GS is caused by autosomal recessive inheritance of inactivating mutations in the* SLC12A3* gene, which encodes a thiazide-sensitive sodium-chloride cotransporter (TSC). Altered TSC protein causes dysfunctional distal convoluted tubule [3]. The typical clinical features of GS are muscle weakness, muscle cramps, periodic paralysis, episodes of tetany, and/or general malaise due to hypokalemia. Convulsions are not common, although it has been reported that convulsions due to severe metabolic alkalosis or hypomagnesemia can occur [4]. Besides GS, there are cases of inherited hypokalemia salt-wasting renal tubular disorders, like SeSAME or EAST syndrome, in which convulsion is one of the chief complaints [5, 6]. SeSAME (seizures, sensorineural deafness, ataxia, mental retardation, and electrolyte imbalance) or EAST (epilepsy, ataxia, sensorineural deafness, and tubulopathy) syndrome is caused by mutations of the K (+) channel* KCNJ10* (*Kir4.1*) gene, which is an autosomal recessive disease [5, 6]. Here, we report the case of a school boy who initially presented with a generalized convulsion that was not associated with hypokalemic paralysis. Subsequent studies revealed that the patient has persistent hypokalemia, metabolic alkalosis, and hyperrenin activity. With genetic analysis, we confirmed that the patient actually had GS, based on the two mutations of the* SLC12A3* gene.

## 2. Case Report

**Figure 1:**
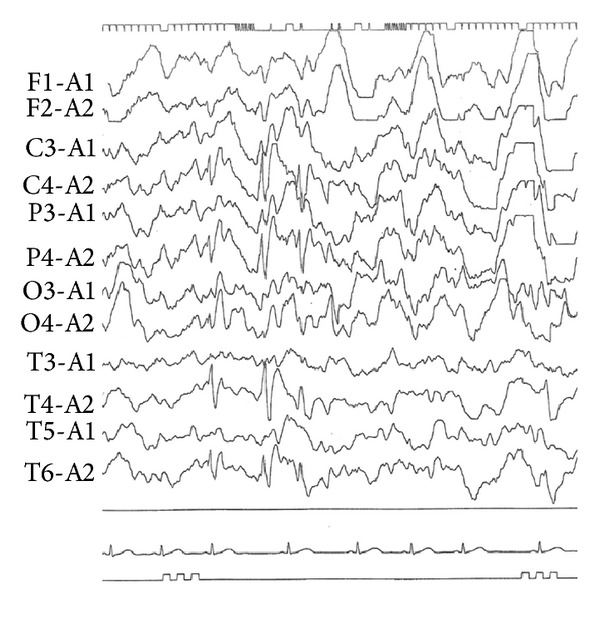
Electroencephalogram shows spike and waves at the C4-A2, P4-A2, T4-A2, and T6-A2 areas. These abnormal findings continue longer than one year.

An 8-year-old Japanese boy developed a generalized convulsion that lasted for 5 min and occurred while he was playing on November 10, 2012. When he was admitted to our hospital by an ambulance, he was conscious and afebrile (temp. 36.3°C). His body height was 120 cm, body weight 21.3 kg, blood pressure 96/42 mmHg, heart rate 92/min, and SpO_2_ 100% at room air. He was neither anemic nor icteric and did not exhibit hepatosplenomegaly, without ataxia, deafness, or mental retardation. No signs showing intracranial hypertension were noted. His past history was unremarkable, except for complaining of muscle weakness after playing football. His family history revealed that his older brother was treated with an anticonvulsant because of attention-deficit hyperactivity disorder. The patient's laboratory findings were white blood cell counts 11,000/*μ*L, Hb 14.1 g/dL, platelet counts 485,000/*μ*L, and serum C-reactive protein 0.04 mg/dL. As summarized in Table 1, his renal function was normal, but the electrolyte assay revealed hypokalemia and blood gas analysis showed the presence of metabolic alkalosis. Endocrine function analyses were within normal with the following values: TSH 3.48 (normal 0.35–4.94) *μ*U/mL, free T3 4.65 (normal 1.73–3.71) pg/mL, free T4 1.41 (normal 0.70–1.48) ng/dL, ACTH 9.5 (normal 7.2–63.3) pg/mL, cortisol 8.2 (normal 4.5–21.1) *μ*g/dL, and aldosterone 14.6 (normal 3.6–24) ng/dL; however, high renin activity was noted (Table 1). The patient was diagnosed with seizure of unknown cause, probably epilepsy, on the basis of the electroencephalogram findings, which showed abnormal spike and waves at the right parietotemporal region (Figure 1). The patient was prescribed potassium L-aspartate (300 mg, two tab/day) for the hypokalemia and carbamazepine (220 mg/day, in two divided doses) to control the seizures. Over the following year, the patient was asymptomatic. However, a year after the hospital admission, the patient was found to still have similar metabolic alkalosis, hypokalemia, hypomagnesemia, high renin activity, and slightly high aldosterone levels (32.0 ng/dL), as well as marked hypocalciuria (the urinary Ca/creatinine ratio was 0.006; normal > 0.2). The hypomagnesemia was associated with extremely high urinary excretion of Mg (7.2 g/L; normal 0.1-0.2 g/L) (Table 1). As summarized in Table 1, FEMg and FECa [7] were dramatically altered to the opposite direction while FEK and TTKG [8] were not significantly changed.

**Figure 2:**
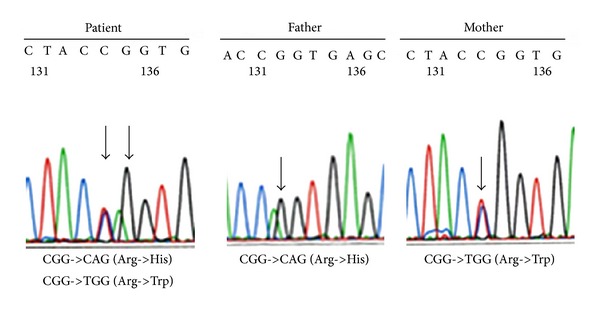
Sequence analysis of the* SLC12A3* gene is shown, which encodes the thiazide-sensitive Na^+^/Cl^−^ cotransporter. The patient had compound heterozygous mutations at codon 642, namely, CGG->CAG (Arg642His) that was inherited from his father and CGG->TGG (Arg642Trp) that was inherited from his mother. His elder brother had a heterozygous mutation in this codon (R642H) (data not shown).

These findings indicated that the patient might have had GS at his initial presentation, which prompted us to examine his* SLC12A3* gene for mutations. For the genetic studies, a written informed consent was obtained from the patient's family in accordance with the Declaration of Helsinki. The institutional review boards of Uji-Tokushukai Medical Center and Hokkaido Graduate School of Medicine approved the genetic diagnosis of the* SLC12A3* gene. Direct sequencing of the* SLC12A3* gene was done as reported previously [9]. Sequencing analysis revealed the presence of compound heterozygous mutations of the codon 642-encoded arginine amino acid in the cytoplasmic tail of the TSC protein: in one gene, the arginine had become a histidine and was inherited from the father; in the other gene, it had become a tryptophan and was inherited from the mother; thus the patient had the R642H/R642W mutation (Figure 2). His older brother had only one of these heterozygous mutations (R642H).

## 3. Discussion

GS is a rare but relatively frequent salt-losing tubulopathy. It should be emphasized that any older children or adult patients who chronically exhibit hypokalemia, metabolic alkalosis, hypocalciuria, and hypomagnesemia should be suspected for GS and appropriately managed. The hypocalciuria and hypomagnesemia in this syndrome are secondary to the high urinary magnesium excretion. At the clinical level, our patient first presented with a convulsion, instead of the more common severe muscle weakness or periodic paralysis. For this reason, he was tentatively diagnosed with epilepsy. While seizures are not common in GS, convulsions due to severe metabolic alkalosis or hypomagnesemia can occur [4, 10], and in such cases, the serum Mg levels are extremely reduced (<1.0 mg/dL) [11, 12]. However, the serum Mg levels of our case were in the range of 1.4–1.8 mg/dL; thus it remains unclear whether hypomagnesemia played a role for the development of the seizure in this patient. In addition, although a rare infancy-onset form of GS has been associated with psychomotor retardation [4], our patient did not exhibit any behavioral disorders, so an alternative explanation for the patient's convulsion may be due to incidental epilepsy, based on the abnormal findings of electroencephalogram. Regarding the nature of his epilepsy, SeSAME/EAST syndrome [5, 6] was unlikely, based on the lack of other clinical features. On the other hand, the sodium-chloride cotransporters can perform a wide variety of physiologic functions in the neurons in the central nervous system, including regulating blood pressure [13]. In fact, idiopathic intracranial hypertension was described in patients with GS possessing altered TSC protein [14, 15]. However, we confirmed that the patient's convulsion was not due to intracranial hypertension.

In terms of the renin-aldosterone system, the majority of GS cases exhibit both hyperrenin activity and hyperaldosteronism, although one rare case report describes GS associated with elevated plasma renin activity and normoaldosteronism [16]. Our patient was initially hyperreninemic with normoaldosteronism; however, a year later, mild hyperaldosteronism became apparent.

In terms of the* SLC12A3* gene mutation, various types of GS-associated* SLC12A3* gene mutations have been reported to date [3, 17].* SLC12A3* gene mutations are particularly prevalent in Japan [18], with one report stating that the GS mutant allele frequency in 1,567 Japanese subjects exceeded 4.8% [19]. As a result, many cases of GS caused by various compound heterozygous or homozygous gene mutations have been identified in Japan [9, 20, 21]. Several mutations in codon 642, which normally encodes an arginine in the cytoplasmic tail of the TSC protein, have been reported: the R642C mutation has been detected in Japanese GS cases [21, 22] and the R642H and R642G have also been reported [3, 23]. However, our patient had different mutations in codon 642, namely, R642H inherited from his father and R642W inherited from his mother, thus yielding a compound heterogeneous mutation. We previously described a case of GS associated with the L858H mutation that initially presented with thyrotoxicosis [20], but the current case was not associated with thyrotoxicosis. After his diagnosis of GS, the present patient has been managed with potassium and Mg supplementation.

In summary, a patient showing persistent hypokalemia and metabolic alkalosis should be tested for GS and, if possible, diagnosed at the genetic level, even if the patient exhibits atypical clinical symptoms.

## Figures and Tables

**Table 1:** Laboratory data obtained on two occasions one year apart.

Data	November 2012	October 2013	References
Serum electrolytes			
s-Na (mEq/L)	139	139	138–146
s-K (mEq/L)	3.2	2.8	3.6–5.1
s-Cl (mEq/L)	97	97	99–108
s-Ca (mg/dL)	10.5	10.0	8.7–10.3
s-P (mg/dL)	NT	3.6	2.9–4.9
s-Mg (mg/dL)	1.4–1.8	1.8	1.8–2.4
Renal function			
s-BUN	17.4	13.3	7.8–18.9
s-creatinine	0.43	0.39	0.64–1.11
FENa (%)	1.2	0.7	<1.0
FEK (%)	12.3	9.8	9.6 (4.6–20.4)∗∗
FEMg (%)	NT	468	1.4 ± 0.6∗
FECa (%)	NT	0.023	0.25 ± 0.2∗
TTKG	NT	9.62	6.0 (4.1–10.5)∗∗
Urinary biochemistry			
u-creatinine (mg/dL)	33.64	33.27	—
u-Na (mEq/L)	130	85	—
u-K (mEq/L)	30.8	23.4	—
u-Cl (mEq/L)	128	92	—
u-Ca (mg/dL)	NT	0.2	—
u-Ca/creatinine	NT	0.006	>0.2
u-Mg (g/L)	NT	7.2	0.1-0.2
Renin activity (ng/mL/hr)	>15.4	47.1	0.2–2.7
Aldosterone (ng/dL)	14.6	32.0	3.6–24
Blood gas^¶^			
pH	7.443	7.459	7.35–7.45
pCO_2_ (mmHg)	45.9	45.3	35–45
pO_2_ (mmHg)	46.1	50.5	80–100
HCO_3_ act (mmol/L)	30.7	31.7	20–26
BE(vt) (mmol/L)	5.7	7.2	−3–+3

FE: functional or fractional excretion; TTKG: transtubular potassium concentration gradient; s: serum; u: urine; NT: not tested; ^¶^venous blood.

Reference values are from ∗Rodríguez-Soriano et al. (Pediatr Nephrol 1990) [7], ∗∗Futrakul et al. (Am J Kidney Dis. 1999) [8].
