# Corrigendum to “Gitelman Syndrome in a School Boy Who Presented with Generalized Convulsion and Had a R642H/R642W Mutation in the *SLC12A3* Gene”

**DOI:** 10.1155/2015/853523

**Published:** 2015-02-28

**Authors:** Shigeru Makino, Toshihiro Tajima, Jun Shinozuka, Aki Ikumi, Hitoshi Awaguni, Shin-ichiro Tanaka, Rikken Maruyama, Shinsaku Imashuku

**Affiliations:** ^1^Division of Pediatrics, Uji-Tokushukai Medical Center, Uji, Kyoto, Japan; ^2^Department of Pediatrics, Hokkaido Graduate School of Medicine, Sapporo, Japan; ^3^Division of Laboratory Medicine, Uji-Tokushukai Medical Center, Uji, Kyoto 611-0042, Japan

“R642H/R642W” in the title of the paper “Gitelman Syndrome in a School Boy Who Presented with Generalized Convulsion and Had a R642H/R642W Mutation in the* SLC12A3* Gene” is wrong and should read “R642H/R642C” so that the title will read “Gitelman Syndrome in a School Boy Who Presented with Generalized Convulsion and Had a R642H/R642C Mutation in the* SLC12A3* Gene.”

The sentence “in the other gene, it had become a tryptophan and was inherited from the mother;” is corrected as “in the other gene, it had become a cysteine and was inherited from the mother;” and the sentence “thus the patient had the R642H/R642W mutation (Figure 2).” is corrected as “thus the patient had the R642H/R642C mutation (Figure 2).”

There is an error in “Figure 2” and it is replaced with a corrected Figure 2.

There is an error in the entire legend of “Figure 2” and it is corrected as mentioned herein.

The sentence “However, our patient had different mutations in codon 642, namely, R642H inherited from his father and R642W inherited from his mother, thus yielding a compound heterogeneous mutation.” is corrected as “However, our patient had different mutations in codon 642, namely, R642H inherited from his father and R642C inherited from his mother, thus yielding a compound heterogeneous mutation.”

At the end, we would like to include an Acknowledgement section as follows: The authors thank Dr. Andrew Phillips, Research Associate, Human Gene Mutation Database Institute of Medical Genetics, Cardiff University, UK, for the reevaluation of sequence analysis.

## Figures and Tables

**Figure 2:**
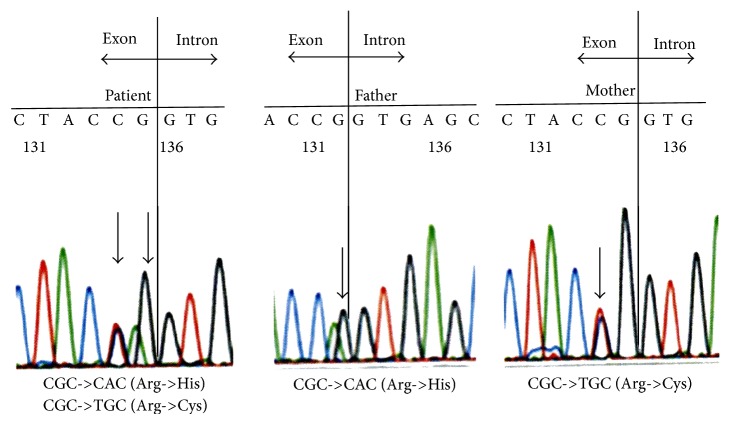
The figure shows genomic rather than cDNA sequence and shows that the substitutions identified were actually c.1925G>A p.Arg642His and c.1924C>T p.Arg642Cys.

